# Diet as a Remedial Agent in the Treatment of Disease

**Published:** 1853-05

**Authors:** 


From the Southern Journal of the Medical and Physical Sciences.

*Diet as a Remedial Agent in the Treatment of Disease.*

It was a quaint maxim of the late Dr. Armstrong that "rest and starvation are the best tonics in the world." According to our own experience, a proper diet is as important to the the successful treatment of disease, as the employment of medicines. So indispensible indeed, is a judicious selection of diet, to the success of any species of medication, that it is difficult in many instances, to determine whether more credit is due to the medicines employed, or to the dietetic management of the case. In fact, no medicine is capable of exerting its beneficial effects, to the fullest extent, and of impressing the system, either locally or generally, to the full measure of its specific virtue, if uuaccompanied with a strict observance of a well regulated diet. It is the besetting sin of a large majority of young practitioners, upon their first entrance into practice, to attach an undue importance to the efficacy of medicine, and to overlook or depreciate the value of dietetic treatment. They are too ready to attribute an *absolute* efficacy to the medicines employed, without a due reference to the condition of the general system, which may promote or *antagonize* the peculiar action of medicines, whether addressed to the system generally or locally. It is doubtless owing to this fact, to some extent at least, that the observations and experience of medical writers have led to such widely different and unsatisfactory conclusions, as to the value of a given remedy in the treatment of certain forms of disease.

The observations of Sir W. Philip, upon the employment of the minute doses of Mercury in the treatment of several chronic affections, would naturally tempt the young practitioner, to ascribe Sir Wilson's remarkable success in the management of these affections, exclusively to the peculiar efficacy of Mercury; but how few ever have employed this medicine with the same happy results? and why? Doubtless, Sir Wilson Philip was not unmindful of the importance of an accurate inquiry into the peculiarities of each individual case subjected to his treatment before, and the influence of habit and diet, in the development and modification of diseased action, but was quite as careful in selecting and enforcing a judicious diet, as he was accurate in his diagnosis, and persevering in the employment of his favorite remedy.

It is a remark often made by older menbers of the profession, that the older they grow, the less medicine they give; which, properly rendered, only means, that as their experience in the management of disease accumulates, they are less disposed to overrate the value of medication, and better prepared to appreciate the importance of a well regulated diet and the influence of favorable conditions and circumstances adapted to each individual case.

Homœopathy, we all know is a delusion, so far as it claims to be a rational system of medicine. But whilst we reject the theory, we must admit, that the strict attention to the dietetic management of disease, so scrupulously enforced by the homœopathic practitioner, is worthy of a candid consideration. If homœopathy has achieved any success in curing disease, it is indebted, almost exclusively, to the superior dietetic rules observed, and to the confidence inspired in the mind of the patient, by the comfortable feelings resulting from a strict regulation of the diet. Indeed, this is the true success of homœopathy in retaining the confidence of many individuals and communities for so long a period of time, and so far outliving the usual term of its kindred delusions.

It is a law in mechanics, that the angle of reflection is equal to the angle of incidence; that action is invariably followed by a corresponding re-action; in morals, that one extreme is sure to beget the opposite; and a similar law holds good in the history of the science and practice of medicine. The "*juste milieu*,"—the judicious middle ground, it is difficult to delineate. The aggregate of the vital forces, in health and disease, in their diversified relations of physical agents, is a complex problem, involving such a variety of propositions, that the human mind will never be competent to its solution. To attain even a comprehensive view of the subject in all its parts, and to be able to assign to each its just importance, in the use of means for the prevention and cure of disease, must necessarily tax to the utmost, the capacities of the most gifted intellect. Between the two extremes of venacavaism and homœopathy, the medical world has been subdivided into a variety of sect, each basing their doctrine and practice upon some favorite text in the Book of Nature, true in itself, but isolated and detached from its just relation to other truths equally weighed, magnified into a superlative importance, and forced to sustain facts not referrible to it, it has become so interwoven with false opinions that in the re-action which sooner or later succeeds, its real merit is depreciated, and for a time grossly neglected. Look, for instance, at the theory of Brown. The value of diaphoresis in the treatment of fevers, is admitted by all; but the rapid resolution of febrile phenomena under the diaphoretic treatment, so fascinated Dr. Brown, with a cetain class of medicines, that he forthwith jumped at the conclusion, that fever is essentially a spasm of the extreme vessels, and discarding almost every other species of medication, with the enthusiasm of a monomaniac, he administrated antispasmodics and diaphoretics in the most extravagant quantities. The result was, that diaphoretic medicines fell into comparative disrepute.

On the other hand, Broussais, struck with the invariable traces, as he imagined, of gastroenteric inflammation, observed in his autopsies of fever cases, conceived the idea that fever is essentially a gastroenteritis; that all that group of phenomena denominated febrile, was directly referrible to intestinal irritation or inflammation; and with this partial and imperfect conception of the essential nature of fever, he framed a theory and system of practice, which for a time, threatened to annihilate every other medical doctrine. The experience of a few years, however, satisfied the profession generally, that the theory itself was inconsistent with the principles of a sound medical philosphy; and that gum water, leeches, and the "*diet absolue*," though valuable in their proper time and place, were wholly unreliable in the management of fevers. But every theory, based upon the "*one idea*," must be necessarily false. Broussais attached too little importance to the efficacy of medicines, and Hahnneman fell into a most grievous blunder, in attributing a potency to medicine, in the ratio of an infinitesimal subdivision of its atoms; and the only merit that can be reasonably claimed for Homœopathy or Broussaism, must be ascribed to the stress laid upon the importance of a strict dietetic management of disease.

Both, however, it seems to us erred to some extent even here. Broussais relied upon the "*diet absolue*," without respect to the nature and symptoms of disease,—in every case, and at every period of the fever, and under all circumstances, gum water was indiscriminately used. The phantom of gastroenteric irritation, continually haunted his imagination, and he was insensible to the cravings of appetite, or the peculiar instincts of the stomach for any particular article of diet or drink, until the supposed inflammatory condition was relieved. The disciples of Hahnneman rely chiefly upon a prescription of such articles of diet or drink, habitually used by their patients, as they suppose favorable to the developement of disease, and partly upon the assumption that certain articles are compatible or otherwise with the legitimate effects of their infinitesimal doses upon the different organs. A globule of lime, operating upon the system through an indefinite period of days, and permeating with metaphysical subtlety the ultimate atoms of the tissues, and stealthily counteracting the poisonous effects of the insidious poison, and driving it from the system, is supposed to be compatible only with a certain quantity and quality of diet. The character of the medicine employed, is made to determine the quality, &c., of the diet, and hence but little discrimination is exercised in selecting the diet, farther than to ensure the fancied compatibility of medicated globules.

It is granted that certain medicines are, therapeutically, more or less compatible with certain rules of diet or drink, and that the efficacy of a remedy is oftentimes increased by a judicious diet.—But the importance of a strict attention to the dietetic management of a disease, is not derived exclusively from this fact. A higher authority than Hippocrates has declared, that "the blood is the life of the flesh;" and as the chemical elements and physiological character of this fluid, are more or less affected and modified in every departure from a healthy condition of the human system, it is certain that the blood must constitute an important element in many diseases, in fevers perhaps invariably. The depraved secretions of the skin and mucous surfaces, and of the organs in the three great cavities, present an assemblage of pathological expressions, that determines to a certain extent, the diagnosis of all febrile diseases; and the value of a given remedy is estimated from its efficacy in altering the secretions of a part, or restoring functional integrity to the different organs. In certain diseases, characterized by an undue proportion of the serum of the blood, and a tendency to serous transudation in the several cavities, we ordinarily employ a class of medicines denominated hydragogues—and in conjunction, or perhaps at a subsequent period of the treatment, we resort to carbonagogues, to depurate the blood, or remove the abnormal accumulation of carbonized materials, and alteratives, or (to use a common expression,) medicines "to improve the secretions," and this constitutes the ordinary routine of practice in a large majority of diseases. But at the same time that medical men, in their every day practice, thus virtually admit that the condition of the blood is the most important element in disease, how little importance is generally attached to the efficacy of diet as a remedial agent in the treatment of their cases.—If it be an object in the treatment of fevers, to remove, by means of different medicines, the noxious and effete materials which had accumulated in the blood, as a "*sine qua non*" to the cure, may we not, at the same time, and in conjunction with a judicious course of medication, accomplish the object more effectually, by prescribing such articles of diet and drink, as will co-operate with the medicines in restoring the blood to its normal condition. Certainly a healthly molecular constitution of blood greatly depends upon the quality of the alimentative matter.

Notwithstanding the unjust odium into which the doctrine of the humoral pathologists has fallen, from the extravagant opinions entertained by the humoralists themselves, and the abuses of mercenary quacks, there is probably more real importance in a correct knowledge of the character of the blood, in health and disease, than the most sanguine humoralist had conceived. Why is it, that in a certain form of disease, the patient should express so strong a preference for a particular description or article of diet or drink above all others? And why should this individual, laboring at a subsequent time under a different species of disease, crave an opposite or different diet or drink? An ague patient for instance, almost invariably relishes oysters, beefsteaks, and other highly nutritious animal diet, rich in fibrinous matter, while the same individual, in an attack of typhoid fever loathes these articles, with almost every other species of solid animal food, and selects butter milk? It is not sufficient to say, that the system in the one case needs a richer blood, than in the other, the fibrinous portion of the circulation requires to be reduced; this is not a satisfactory statement even of the fact itself. Why should an individual, who was last week eating freely of ham and beef, and to-day the subject of typhoid fever, feel a disgust at the bare suggestion of such a diet, and crave an article of diet and drink which perhaps he has no relish for in health? To say that the sense of taste is modified or impaired by the fever, or that such an article is more grateful to the stomach of a typhoid fever patient, is the mere statement of the fact, but offers no satisfactory solution of the cause. An infant of twelve months has been the subject of cholera infantum for the last four months, and is reduced to an extreme of anæmia and emaciation that is scarcely compatible with the phenomena of organic life. Week after week, it has been subjected to another and a different course of medication and diet, until medicines have almost entirely ceased to exert any beneficial influence upon the stomach and bowels; food, of the mildest and most digestible duality, is passed through the bowels in an undigested state, and the medical attendant concludes that the functions of digestion and assimilation are so far impaired, that the little patient must inevitably die of inanition. At this juncture he casually learns from the nurse that for several days or weeks past, the child has been in the habit of crying at the sight of pine apple cheese, an article of diet, perhaps of all others, the most unsuitable for an infant, and difficult of digestion by even the strongest stomach. As an extremely hazardous experiment, but justifiable under the circustances of the case, he consents that the little patient should be freely indulged with the cheese, and forthwith a stomach, which for months past could not digest the mildest, and, under ordinary circumstances, articles of diet the most easily assimilated, readily digests this strong diet, the bowels begin to improve, and the patient, to the suprise of all parties, is suddenly snatched from the very jaws of death, and rapidly restored to health and flesh. Now, this is no fancy case, and the experience of many physicians could furnish many cases similar, if not more remarkable even, than the one described.

There are two points of much interest in the above case, which, however, we do not claim to solve to the satisfaction of the reader: Why did the child crave the food in preference to any other species of aliment, and how could a stomach enfeebled by constant medication, and impaired by a protracted disease, readily digest and assimilate an article of diet ordinarily so difficult of digestion? The fact is certainly diametricelly opposed to the commonly received doctrines and practice of the medical profession, and strangely inconsistent with our general experience. There seems to be a species of instinct in such cases, similar to what we observe in animals, as they select certain species of herbs and grass, and reject others. But where does this instinct reside, in the stomach, the bowels, the brain, or the blood? What faculty instigates the suggestion, that this, or that species of nutrative matter is required by the system? So far as the case of the child is concerned, it could not have been because it had been habitually fed upon cheese, or because the cheese, as a therapeutic agent, possessed any efficacy in curing cholera infantum. In a great majority of such cases, in the earlier stages at least, and even in the ordinary condition of infantile health, no article of diet would be more likely to disturb the stomach and bowels.

The only rational conclusion we can deduce from such facts is, that this instinct, (if it be lawful so to term it,) is an expression of the elective affinities of the blood for certain ultimate or proximate elements contained in particular kinds of alimentative material, which are required by the blood, to restore the defective or imperfect protein element, and establish the normal relation of its constituent parts. In the case of the child with cholera infantum, had the cheese been withheld, in all probability, death would have been inevitable. The physiological character of the blood had been so changed and modified by disease, and the failure of the organs of assimilation to supply the elements requisite to a normal hematosis, that the vital forces were on the eve of suspension. Whether hematosis be the function of the inner tunic of the blood vessels, or endangium, as Dr. Dewees terms it, or an effect of the chemicovital action of atmospheric air upon the chyle, through the medium of the lungs, aided by the assimilating force of the blood globules, is a physiological problem yet to be solved; recent observations, however, seem to have established the fact, that there is in the circulation of every individual in health, an amount of imperfectly assimilated chyle, varying in quantity in different individuals subject to elimination by the several depurating organs, as unsuited to the molecular regeneration of the tissues, and yet not strictly effete matter, as the products of the destructive metamorphosis of the tissues. Now the question is, whether the nondescript chemical element results from incomplete or defective hematosis, or imperfect chylification. In many cases, it may be, that in the absence of a supply of the proper elements, from primary assimilation, the *chemico-vital elective* affinities of the blood are competent to appropriate this imperfectly elaborated nutritive material, and even to re-arrange and combine the products of secondary assimilation, and restore the blood to a comparatively normal condition.

[*To be continued.*]

